# Comparison of Cardiovascular Response to Lower Body and Whole Body Exercise Among Sedentary Young Adults

**DOI:** 10.7759/cureus.45880

**Published:** 2023-09-24

**Authors:** Anita Kumari, Swati Sinha, Amita Kumari, Anup Kumar D Dhanvijay, Sanjeet Kumar Singh, Himel Mondal

**Affiliations:** 1 Physiology, All India Institute of Medical Sciences, Deoghar, IND; 2 Physiology, Bhagwan Mahavir Institute of Medical Sciences, Pawapuri, IND; 3 Pathology, All India Institute of Medical Sciences, Deoghar, IND

**Keywords:** chatgpt, exercise physiology, blood pressure, heart rate, exercise, treadmill, bicycle ergometer, young adult males, whole-body exercise, lower body exercise

## Abstract

Background

Cardiovascular responses to exercise are essential indicators of cardiovascular health and fitness. Understanding how different types of exercise, such as lower-body and whole-body exercises, impact these responses is crucial for designing effective fitness programs and assessing cardiovascular function.

Aim

This study aimed to compare the cardiovascular response of young adults during lower-body exercise using a bicycle ergometer and whole-body exercise on a treadmill.

Methods

Thirty-two healthy young adults participated in this study. Each participant completed two exercise sessions on separate days: lower-body exercise on a bicycle ergometer with a fixed cadence of 60 rpm with a breaking resistance of 1.75 kg and whole-body exercise on a treadmill with a speed of 1.7 mph and a 10% grade. Heart rate (HR), systolic blood pressure (BP), and diastolic BP were measured at rest and immediately after a three-minute exercise. Data were analyzed using paired t-tests to compare the cardiovascular responses between the two exercise modalities.

Results

A total of 17 male and 15 female young adults with a mean age of 20.87±1.43 years participated in the study. The male and female participants had similar ages (p =0.56) and body mass indexes (p = 0.1). There was a higher HR (129.16±2.67 versus 150.87±3.23, p<0.0001) and systolic BP (127.29±2.34 versus 144.9±4.16, p<0.0001) and lower diastolic BP (68.97±2.41 versus 62.97±2.31, p<0.0001) in whole body exercise on treadmill compared to lower body exercise in bicycle ergometer. The effect size was large enough as Cohen's d was 7.33, 5.13, and 2.54 for HR, systolic BP, and diastolic BP, respectively.

Conclusion

In sedentary young adults, treadmill exercise led to higher HR, systolic BP, and lower diastolic BP than bicycle ergometer exercise. Increased muscle recruitment might result in higher energy expenditure, increasing the HR and systolic BP to deliver oxygen and nutrients to the working muscles. Further research is needed to understand the mechanisms and long-term implications for precise exercise recommendations and better cardiovascular health management.

## Introduction

Cardiovascular health is an indicator of overall well-being. Regular physical exercise plays a pivotal role in the maintenance and enhancement of cardiovascular health. Understanding how different types of exercise affect cardiovascular responses is of paramount importance, as it informs the design of effective fitness regimens and aids in the assessment of cardiovascular function. Among the available exercise modalities, lower-body exercise and whole-body exercise represent two fundamental categories that engage distinct muscle groups and physiological systems [1,2].

Lower body exercise, often performed on a stationary bicycle ergometer, predominantly involves using the lower extremities. In contrast, whole-body exercise, such as running or walking on a treadmill, engages both the upper and lower body and necessitates a higher degree of coordination and energy expenditure [3]. While the benefits of regular exercise on cardiovascular health are well-established, the extent to which these two modalities elicit differential cardiovascular responses remains an area of scientific interest [4].

The cardiovascular response to exercise is characterized by dynamic changes in heart rate, blood pressure, and oxygen consumption, among other parameters. These responses are influenced by various factors, including exercise intensity, duration, and the muscle groups involved [5,6]. Additionally, individual fitness levels and physiological adaptations further contribute to the variability in cardiovascular responses to exercise.

Sedentary individuals and athletes exhibit distinct cardiovascular responses during exercise due to differences in their baseline fitness levels and physiological adaptations. Sedentary individuals typically have higher resting heart rates, which rise rapidly during exercise, along with pronounced increases in blood pressure and cardiac output. Their lower aerobic capacity means they may reach their limits sooner and experience longer recovery times [7]. In contrast, athletes benefit from lower resting heart rates, controlled heart rate responses, stable blood pressure, and more efficient oxygen delivery to muscles, thanks to their conditioning [8].

A study by Turley and Wilmore reported that the cardiovascular response does not depend on exercise modality [9]. Exercise on a cycle ergometer showed a more considerable change in functional capacity when compared to treadmill exercise [10]. Cycling resulted in notably elevated cardiopulmonary reactions compared to treadmill exercise in peripheral vascular disease patients [11]. In contrast, increased cardiovascular reactions were observed in exercise on treadmills among Nigerian male hypertensive participants when compared with bicycle ergometers [12]. Furthermore, in a diagnostic test, exercise on both a cycle ergometer and treadmill generates a similar response [13]. In obese women, responses were different and it was concluded that walking is more convenient for obese women [14]. A study with Indian young adults showed a higher cardiovascular response and reported a higher response in whole-body exercise on a treadmill [15].

With this context, this study aimed to compare young adults' cardiovascular responses during lower-body exercise on a bicycle ergometer and whole-body exercise on a treadmill.

## Materials and methods

Study setting

The study was conducted in the Department of Physiology at the Indira Gandhi Institute of Medical Sciences (IGIMS) in Patna, Bihar, India after obtaining institutional ethics clearance (567/ACAD 22/06/2016).

Participant recruitment

We used a convenience sample for this study. A total of 40 apparently healthy sedentary young adults in the age group of 18-25 years were recruited for the study. The procedure of the study was thoroughly explained to potential participants and informed written consent was obtained from each individual. The inclusion criteria for participants were healthy young adults within the age group of 18-25 years. Exclusion criteria included individuals who were actively engaged in regular gym training or exercise, had a known cardiac or respiratory disorder, had any known medical or surgical illness, or had a physical disability. Additionally, participants who were taking any medications were excluded from the study.

Familiarization sessions

Prior to the experimental trial, which involved the recording of study variables, participants underwent four to six familiarization sessions. During these sessions, participants were provided with instructions and demonstrations on maintaining an upright posture while walking on the treadmill, emphasizing not holding the side rails or leaning forward to preserve the body's center of mass. For the bicycle ergometer exercise, participants were taught to lightly hold the handlebars without squeezing them, thereby minimizing isometric contractions.

Materials used

A motorized treadmill was employed as one of the exercise modalities in this study. The treadmill allowed for controlled walking exercise at specified speeds and inclinations, facilitating a standardized exercise regimen. The speed was 1.7 mph at 10% inclination. A bicycle ergometer was utilized as the next mode of exercise modality. Participants pedaled on the ergometer at a predefined rate, enabling a consistent and controlled exercise routine. The speed was 60 rpm with a breaking resistance of 1.75 kg [15]. Pulse oximeters were employed to monitor heart rate continuously during exercise sessions. These devices measured the real-time heart rate. An aneroid sphygmomanometer was used to measure blood pressure. An expert clinician measured the blood pressure in resting and immediately after exercise.

Study process

**Figure 1:**
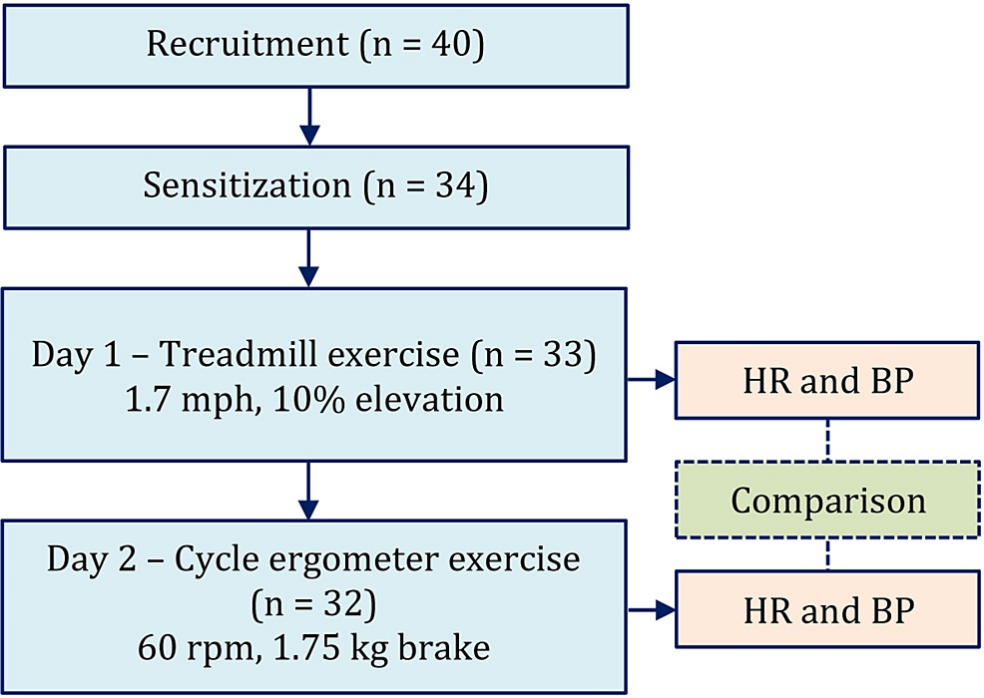
Study participant numbers and brief study procedure n = number, mph = mile per hour, rpm = rotation per minute, HR = heart rate, BP = blood pressure. As one participant was lost on the second day, the final data analysis was for 32 participants.

On the first day, the subjects were exercised on the treadmill after a 1-hour resting at 1.7 mph speed and 10% elevation. On the second day, they exercised on the cycle ergometer at 60 rpm with a 1.75 kg brake. As the exercises were carried out a day apart, there is less chance of influencing one session on other sessions. The brief process is shown in Figure 1.

Data analysis

The data were expressed in mean and standard deviation. The heart rate and blood pressure were compared between the sessions by paired t-test. The parameters between males and females were tested by unpaired t-test. The GraphPad Prism 9.5.0 (GraphPad Software, Inc., Boston, Massachusetts, United States) was used for statistical analysis.

## Results

A total of 17 male and 15 female young adults participated in the study. The age, height, weight, and BMI are shown in Table 1. The age was similar in males and females. Although the height and weight were higher in males, the BMI between them was not significantly different.

**Table 1:** Age, height, weight, and body mass index of participants BMI: Body mass index *Statistically significant p-value of unpaired t-test

Variable	Overall (n = 32)	Male (n = 17)	Female (n = 15)	p-value
Age (years)	20.87±1.43	20.71±1.36	21.07±1.54	0.56
Height (cm)	163.61±8.14	166.86±7.74	159.66±6.96	0.03*
Weight (kg)	68.61±10.64	73.54±10.19	62.62±7.93	0.003*
BMI (kg/m^2^)	25.61±3.61	26.49±4.18	24.53±2.5	0.1

The resting HR and systolic and diastolic BP and the corresponding values immediately after exercise are shown in Table 2.

**Table 2:** Heart rate and blood pressure at resting and immediately after three-minute exercise HR = Hear rate, BP = blood pressure, mmHg = millimeter of mercury

	Cycle ergometer	Treadmill	p-value
Resting			
HR (bpm)	72.94±1.65	73.03±03	0.75
Systolic BP (mmHg)	113.81±3.36	114.84±2.91	0.28
Diastolic BP (mmHg)	74.26±3.13	74.32±3.06	0.82
Exercise			
HR (bpm)	129.16±2.67	150.87±3.23	<0.0001
Systolic BP (mmHg)	127.29±2.34	144.9±4.16	<0.0001
Diastolic BP (mmHg)	68.97±2.41	62.97±2.31	<0.0001

There was a higher HR (150.87±3.23 versus 129.16±2.67 bpm) and systolic BP (144.9±4.16 versus 127.29±2.34 mmHg) and lower diastolic BP (62.97±2.31 versus 68.97±2.41 mmHg) in whole-body exercise on the treadmill compared to lower-body exercise on the bicycle ergometer. The effect size was large enough as Cohen's d were 7.33, 5.13, and 2.54 for HR, systolic BP, and diastolic BP, respectively.

## Discussion

The observed differences in cardiovascular responses between whole-body exercise on a treadmill and lower-body exercise on a bicycle ergometer can be attributed to a combination of physiological and biomechanical factors. When individuals engage in treadmill exercise, they involve a larger muscle mass, including the upper body, for balance and coordination [16]. This increased muscle recruitment results in higher energy expenditure, necessitating a greater HR to deliver oxygen and nutrients to the working muscles. Furthermore, treadmill exercise places a higher cardiovascular demand on the body due to the simultaneous engagement of both upper and lower body muscles, leading to a higher HR and systolic BP. The redistribution of blood flow, with increased vasodilation in active muscles and vasoconstriction in less active areas, may contribute to the lower diastolic BP observed during treadmill exercise [17].

Exercise intensity also plays a crucial role, as treadmill running typically involves higher intensity than stationary cycling, resulting in more pronounced cardiovascular stress. Additionally, neural control mechanisms such as central command and the baroreflex respond to these physiological changes, influencing HR and BP regulation during exercise [18]. Individual variations in fitness levels and genetics can further modulate these responses. Overall, these findings highlight the intricate interplay of factors that govern cardiovascular responses to different modes of exercise among sedentary young adults [19].

The findings of the present study align with some of the previous research while also contrasting with others. For instance, the study's observation of higher cardiovascular responses is consistent with the findings reported by Abiodun et al. and Lafortuna et al. [12,14]. However, the present study's results contradict the findings reported by Gerlach et al. and Tuner et al. [10, 11]. The underlying reason may be attributed to age, ethnicity, and physiological characteristics [20].

For individuals, the current study emphasizes the importance of choosing exercise modalities. Those seeking a vigorous workout may opt for whole-body exercises on a treadmill, while individuals with cardiovascular concerns might consider lower-impact options like stationary cycling. Healthcare providers can use this knowledge to design safer and more effective exercise prescriptions for patients. Furthermore, these findings can motivate individuals to adhere to their exercise routines by providing insights into the intensity and benefits of various activities.

The study, despite its valuable contributions, has some limitations that should be taken into account when interpreting the result. These limitations include potential issues related to sample size and diversity, variations in exercise protocols, and the lack of long-term analysis. Additionally, environmental factors, measurement equipment accuracy, and selection biases may have influenced the results. Furthermore, it was a single study and its results may not be generalized.

## Conclusions

In this study, we found that in sedentary young adults, there was higher HR, higher systolic BP, and lower diastolic BP during whole-body exercise on a treadmill in comparison to lower-body exercise on a bicycle ergometer. These observations, supported by substantial effect sizes, underscore the importance of considering individualized exercise prescriptions based on fitness goals, health status, and specific cardiovascular conditions. While the study sheds light on these acute responses, further research is warranted to explore the underlying mechanisms and long-term implications, allowing for more precise exercise recommendations and improved cardiovascular health management.
